# Supplementary material for: Amaranth: Enhanced Single-Cell Transcript Assembly via Discriminative Modeling of UMI Reads and Internal Reads
Source: bioRxiv. 2025 Nov 27:2025.11.24.690228. Preprint. [Version 1] doi: 10.1101/2025.11.24.690228 (PMC12676538; doi:10.1101/2025.11.24.690228)
Supplement: Supplement 1 [file media-1.pdf]

Supplement information

S1 Default parameters of Amaranth for Smart-seq3

- `--min_umi_reads_bundle` (*int*, default: 1): Bundle (gene locus) with less UMI reads than this threshold will be ignored.
- `--min_umi_ratio_bundle` (*float*, default: 0.0): Bundle (gene locus) with lower UMI reads ratio than this threshold will be ignored.
- `--both_umi_support` (*flag*, default: off): If set, a bundle needs to satisfy both UMI support thresholds (`--min_umi_reads_bundle` and `--min_umi_ratio_bundle`). Otherwise, satisfying either is sufficient.
- `--min_umi_reads_start_exon` (*int*, default: 1): Minimum number of UMI reads supporting the first exon in a valid transcript.
- `--remove-reteind-intron` (*flag*, default: on): Remove retained introns. To disable this option, use `--no-remove-reteind-intron`.
- `--no-remove-reteind-intron` (*flag*, default: off): Do not remove retained introns.
- `--remove-pcr-duplicates` (*int*, default: 0): Option `0`: do not remove; Or option `1`: remove PCR duplicates with identical alignment coordinates and CIGAR string.
- `--max-ir-part-ratio-v` (*float*, default: 0.5): The ratio threshold of retained node to skip edge for partial introns. If greater than threshold, consider true transcript.
- `--max-ir-part-ratio-e` (*float*, default: 0.5): The ratio threshold of retained node's edge to skip edge for partial introns. If greater than threshold, consider true transcript.
- `--max-ir-full-ratio-v` (*float*, default: 1.0): The ratio threshold of retained node to skip edge for full introns. If greater than threshold, consider true transcript.
- `--max-ir-full-ratio-e` (*float*, default: 0.5): The ratio threshold of retained node's edge to skip edge for full introns. If greater than threshold, consider true transcript.

- `--max-ir-full-ratio-i` (*float*, default: 10.0): The ratio threshold of retained node to its own edge for full introns. If greater than threshold, consider true retention.
- `--max-ir-umi-support-full` (*int*, default: 3): The number of minimal UMI reads to support a partial exon rather than a full intron retention. If lower than the threshold, consider true retention.
- `--max-ir-umi-support-partial` (*int*, default: 5): The number of minimal UMI reads to support a partial exon rather than partial intron retention. If lower than the threshold, consider true retention.
- `--cb-supp-ratio` (*double*, default: 0.3): For meta-assembly only, minimum ratio of exons in a transcript that is supported by a cell's barcode (CB) to assign this transcript to this cell. Only spliced reads are counted.
